# Racial Differences in Screening Eligibility by Breast Density After State-Level Insurance Expansion

**DOI:** 10.1001/jamanetworkopen.2025.25216

**Published:** 2025-08-05

**Authors:** Mattia A. Mahmoud, Sarah Ehsan, Sara P. Ginzberg, Susan M. Domchek, Katherine L. Nathanson, Emily F. Conant, Despina Kontos, Jinbo Chen, Christine E. Edmonds, Oluwadamilola M. Fayanju, Anne Marie McCarthy

**Affiliations:** 1 Department of Biostatistics, Epidemiology, & Informatics, Perelman School of Medicine, University of Pennsylvania, Philadelphia; 2 Department of Surgery, Perelman School of Medicine, University of Pennsylvania, Philadelphia; 3 Department of Medicine, Perelman School of Medicine, University of Pennsylvania, Philadelphia; 4 Abramson Cancer Center, Penn Medicine, Philadelphia, Pennsylvania; 5 Department of Radiology, Perelman School of Medicine, University of Pennsylvania, Philadelphia

## Abstract

**Importance:**

Women with dense breasts have elevated risk of false-negative mammograms and may benefit from supplemental screening.

**Objective:**

To assess potential outcomes of Pennsylvania’s law mandating insurance coverage for supplemental breast cancer screening among Black and White women.

**Design, Setting, and Participants:**

This cross-sectional study included Black and White women, aged 40 to 74 years, without prior breast cancer or known *BRCA1/2* variants who underwent mammography screening at a large urban academic health system from January 2015 to December 2021, with cancer outcomes ascertained through December 2022. Data analysis was conducted from June 2023 to April 2025.

**Exposures:**

Dense breasts; the law mandates insurance coverage for women with extremely dense breasts or those with heterogeneously dense breasts plus a greater than 20% lifetime breast cancer risk by risk models.

**Main Outcomes and Measures:**

The main outcomes were odds of eligibility for supplemental coverage and of a false-negative mammogram.

**Results:**

A total of 68 478 women (38 397 Black women [median (IQR) age, 57 (49-64) years] and 30 081 White women [median (IQR) age, 58 (49-65) years]) were used for the analysis. Fewer Black women had extremely dense breasts (561 [2.1%] vs 1464 [5.8%]; *P* = .02) and greater than 20% lifetime risk of breast cancer identified (257 [0.7%] vs 1905 [6.4%]; *P* = .04) compared with White women. Consequently, Black women were less likely to meet the eligibility criteria for supplemental screening (523 [1.6%] vs 2081 [8.4%]; *P* = .02). The criteria showed lower sensitivity but higher specificity for detecting false-negative mammograms in Black compared with White women in one round of screening. Using heterogeneously or extremely dense breasts alone would detect more false negatives but with significantly more women recommended for magnetic resonance imaging.

**Conclusions and Relevance:**

In this study of 68 478 screening mammograms among Black and White women from 2015 to 2021, retrospectively applying criteria for supplemental screening based on heterogeneously or extremely dense breast density and lifetime risk had limited ability to identify women at risk for a false-negative mammogram. Given lower density and lifetime risk estimates, few Black women met criteria for insurance coverage in Pennsylvania, and the criteria had poor sensitivity for identifying Black women with false-negative mammograms. Additionally, using the current breast density criteria for magnetic resonance imaging may not accurately reflect breast cancer risk in Black women.

## Key Points

**Question:**

How does eligibility for supplemental breast cancer screening based on breast density differ by race?

**Findings:**

In this cross-sectional study of 68 478 Black and White women who underwent mammography, fewer Black women had extremely dense breasts or high lifetime breast cancer risk, making them less likely than White women to be eligible for insurance coverage of supplemental screening. The criteria for coverage identified fewer Black women with false-negative mammograms than White women.

**Meaning:**

The findings of this study suggest that policies for insurance coverage of supplemental screening based on breast density may have limited ability to improve early detection for Black women.

## Teaser

This cross-sectional study evaluates how a Pennsylvania law mandating insurance coverage for supplemental breast cancer screening affects Black and White women who have undergone mammography.

## Introduction

Breast density, the amount of fibroglandular tissue relative to fatty tissue in the breast, is categorized in clinical practice using the Breast Imaging and Reporting Data System (BI-RADS), which involves a radiologist’s visual assessment of a women’s density level into 1 of 4 categories of increasing density: entirely fatty (BI-RADS A), scattered fibroglandular tissue (BI-RADS B), heterogeneously dense (BI-RADS C), and extremely dense (BI-RADS D).^1^ Women with dense breasts (heterogeneously or extremely dense) have up to a 5-fold higher risk of breast cancer compared with women with nondense breasts.^2,3^ Worse, they also have higher rates of false-negative mammograms, as dense breasts can mask tumors on screening, reducing the sensitivity of mammography.^4^ As such, mammography facilities were recently required to inform women about their breast density levels under the Mammography Quality Standards Act (MQSA).^4^ Additionally, women with dense breasts may benefit from supplemental screening with ultrasonography or breast magnetic resonance imaging (MRI), which have higher sensitivity for detecting invasive cancers than mammography.^4^ The Dense Tissue and Early Breast Neoplasm Screening (DENSE) trial showed that women with extremely dense breasts who underwent one round of MRI screening after a negative mammogram had 50% lower false-negative rates than women in the mammography-only group.^4^

Advocacy groups in the United States have successfully lobbied for both federal and state legislation mandating reporting of breast density level to patients, so that they may consider supplemental screening, and a total of 39 states have passed laws mandating insurance coverage of supplemental screening for women with dense breasts.^5^ In response to the growing evidence of the benefits of supplemental MRI, Pennsylvania passed Act 1 of 2023 on May 1, 2023,^6^ which mandates insurance coverage of supplemental screening for women at increased risk of breast cancer, as defined by several criteria, including dense breasts or greater than 10% lifetime risk of breast cancer (Box).

Box. **Pennsylvania Act 1 of 2023 Supplemental Breast Screening Eligibility Criteria**

**High-risk factors covered by Act 1**

- Personal history of breast cancer
- Family history of breast cancer
- Personal history of abnormal breast screenings
- Extremely dense breast tissue
- Heterogeneously dense breast tissue with 1 additional risk factor, including:
  - >20% Lifetime risk of breast cancer according to risk assessment tools
  - Personal history of *BRCA1* or *BRCA2* gene variants
  - First-degree relative with a *BRCA1* or *BRCA2* gene variant but not having had genetic testing herself

Although the support provided by this bill will increase screening access to women in the general population, the outcome of these laws on racial disparities in cancer detection after supplemental screening is unclear. On average, Black women have a lower overall BI-RADS categorized breast density than White women based on the subjective BI-RADS grading, due to the observed association between body mass index (BMI) and breast density.^7,8^ BMI has been inversely associated with percentage density measures, partly as women with higher BMIs tend to have larger, more nondense breasts.^7^

The purpose of our study was to estimate the potential differences in eligibility for the Pennsylvania supplemental screening criteria based on breast density between Black and White women, given existing differences in the prevalence of dense breasts. We retrospectively applied the breast density eligibility criteria for insurance coverage of supplemental screening in Pennsylvania to women undergoing mammography screening at our large urban hospital to compare the proportion of Black and White women who would meet these criteria. Additionally, we examined the sensitivity and specificity of Pennsylvania law criteria vs breast density alone for identifying women who had a subsequent false-negative mammogram. It is important to proactively assess how new policies might affect different patient populations to help ensure that emerging practices do not create or worsen existing disparities in breast cancer.

## Methods

We conducted a retrospective cross-sectional analysis among women without prior breast cancer or known *BRCA1/2* variants who underwent routine screening mammography and completed risk factor questionnaires from January 2015 to December 2021 and had a full year of follow-up to focus our study on women with dense breasts (Figure). The study was compliant with the Health Insurance Portability and Accountability Act and approved by the University of Pennsylvania institutional review board, which granted a waiver of informed consent because this study was a secondary analysis of data obtained during routine clinical care. The Strengthening the Reporting of Observational Studies in Epidemiology (STROBE) checklist was reviewed, and the article was drafted in accordance with these guidelines.^9^

**Figure.**
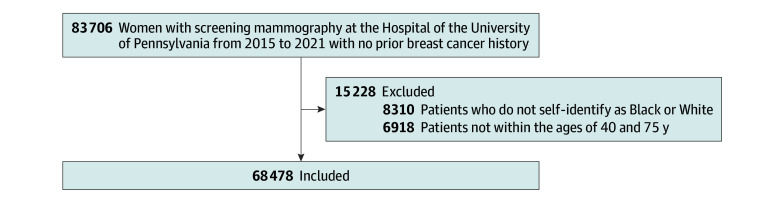
Inclusion and Exclusion Criteria

Our study population included Black or White women (including Hispanic women) based on self-reported race noted in the electronic health record, between the ages of 40 and 74 years, for a total of 68 478 women. For each mammogram, breast density was characterized according to American College of Radiology BI-RADS density categories: almost entirely fatty, scattered areas of fibroglandular density, heterogeneously dense, and extremely dense. According to Pennsylvania law, women can be considered eligible for supplemental screening if they are high risk as defined by several criteria (Box). For our study, we focused on eligibility criteria based on breast density or Gail risk greater than 20% among women with no personal history of breast cancer. We defined women as meeting eligibility criteria if they had (1) extremely dense breasts or (2) heterogeneously dense breasts based on BI-RADS categorization and greater than 20% lifetime risk of breast cancer. Lifetime risk was estimated using the Breast Cancer Risk Assessment Tool (BCRAT).^10^

A false-negative mammogram was defined as a cancer diagnosis within 1 year of a nonactionable screening mammogram (initial BI-RADS assessment of negative [1] or benign [2]). A true-positive mammogram was calculated as a cancer diagnosis within 1 year of a positive mammogram (initial screening BI-RADS assessment of 0, with final BI-RADS at diagnostic recall assessments of 3, 4, or 5). The cancer detection rate (CDR) was defined as the number of true-positive mammograms per total number of mammograms. The interval cancer rate was defined as the number of false-negative mammograms among the total number of mammograms. Both rates were expressed per 1000 screening mammograms.^11^ We report the performance metrics (true positive [TP], CDR, false negative [FN], and interval cancer rate) overall; by density, lifetime risk, and insurance coverage; and stratified by race, as typically done using benchmark guidelines in the Breast Cancer Surveillance Consortium.^12,13^

### Statistical Analysis

Characteristics of Black and White women were summarized by frequency and percentage or mean and SD values of BI-RADS density, age, and eligibility for insurance coverage. We also examined the sensitivity and specificity of both insurance coverage criteria based on the Pennsylvania law (BI-RADS heterogeneously dense and BCRAT >20% or BI-RADS extremely dense) as well as using BI-RADS breast density (heterogeneously or extremely dense breasts vs entirely fatty or scattered fibroglandular tissue) with respect to false-negative breast cancer among women with negative mammograms.

We tested for associations of eligibility according to the Pennsylvania law and risk of a false-negative mammogram, adjusting for age and BI-RADS density and stratified by race, among women with negative mammograms, using logistic regression. We also tested the association between race and the odds of eligibility, adjusted for age and BI-RADS density. For each analysis, women with missing BCRAT lifetime risk percentages were considered low risk, reflecting how they would be clinically treated (primary analysis), and a complete case analysis was conducted with women who had an available BCRAT score (secondary analysis). As a sensitivity analysis, we stratified the analysis by pre– and post–COVID-19 years (2015-2019 vs 2020-2021). Statistical tests were 2-sided with an α level of .05 considered significant. All data analyses and management were performed using RStudio version 1.4.1106 (R Foundation for Statistical Computing).

## Results

A total of 68 478 screening mammograms collected over the study period were included in the analysis, with 38 397 mammograms among Black women and 30 081 mammograms among White women (Table 1). Median (IQR) age was similar for Black and White women (57 [49-64] years vs 58 [49-65] years; *P* = .82). Overall, White women had higher interval cancer rates than Black women (1.2 [95% CI, 0.8-1.6] vs 0.4 [95% CI, 0.3-0.7] per 1000; *P* = .02) while cancer detection rates (5.9 [95% CI, 5.1-6.9] vs 5.6 [95% CI, 4.9-6.4] per 1000; *P* = .49) were similar between the 2 groups.

**Table 1.** Breast Density, BCRAT Lifetime Risk, and Eligibility for Insurance Coverage of Supplemental Screening in Pennsylvania Among Women With Mammography Screening at the Hospital of the University of Pennsylvania, 2015-2021

Characteristic	All (N = 68 478)										*P* values comparing metrics by race			
	White women (n = 30 081)					Black women (n = 38 397)					TP	CDR	FN	ICR
	No (%)	TP, No.^a^	CDR per 1000 (95% CI)^b^	FN, No.^c^	ICR per 1000 (95% CI)^d^	No (%)	TP, No.^a^	CDR per 1000 (95% CI)^b^	FN, No.^c^	ICR per 1000 (95% CI)^d^				
Total screens	30 081 (100.0)	178	5.9 (5.1-6.9)	35	1.2 (0.8-1.6)	38 397 (100.0)	215	5.6 (4.9-6.4)	17	0.4 (0.3-0.7)	.57	.49	.02	.02
Age, median (IQR), y^e^	58 (49-65)	NA	NA	NA	NA	57 (49-64)	NA	NA	NA	NA	NA	NA	NA	NA
BI-RADS Density														
Almost entirely fatty	1779 (5.3)	4	2.3 (0.9-5.8)	0	0 (0.0-2.1)	5772 (15.1)	18	3.1 (1.9-4.9)	0	0 (0.0-0.6)	.12	.08	NA	NA
Scattered fibroglandular	15 862 (53.7)	106	6.7 (5.5-8.1)	9	0.6 (0.3-1.1)	24 663 (63.6)	150	6.1 (5.2-7.1)	8	0.3 (0.2-0.6)	.21	.31	.52	.41
Heterogeneously dense	10 976 (35.2)	55	5.0 (3.9-6.5)	19	1.7 (1.1-2.7)	7401 (19.2)	45	6.1 (4.6-8.1)	9	1.2 (0.6-2.3)	.14	.28	.03	.05
Extremely dense	1464 (5.8)	3	2.0 (0.7-6.4)	7	4.8 (2.3-9.8)	561 (2.1)	2	3.6 (0.9-12.5)	0	0 (0.0-6.6)	.65	.52	<.001	<.001
>20% Lifetime risk based on the BCRAT Model														
No	22 991 (76.4)	121	5.3 (4.4-6.3)	20	0.9 (0.6-1.3)	32 374 (84.3)	182	5.6 (4.9-6.4)	14	0.4 (0.3-0.7)	.43	.66	.13	.12
Yes	1905 (6.4)	16	8.4 (5.2-13.5)	8	4.2 (2.1-8.2)	257 (0.7)	1	3.9 (0.7-20.4)	0	0 (0.0-14.4)	.03	.03	<.001	<.001
Missing	5185 (17.2)	32	6.2 (4.4-8.7)	3	0.6 (0.2-1.8)	5766 (15.0)	31	5.4 (3.8-7.6)	7	1.2 (0.6-2.5)	.75	.64	.14	.15
Meets PA insurance coverage criteria, primary analysis^f^^,^^g^														
No	27 754 (92.3)	156	5.6 (4.8-6.6)	24	0.9 (0.58-1.29)	37 776 (97.6)	212	5.6 (4.9-6.4)	17	0.5 (0.3-0.7)	.88	.95	.19	.12
Yes	2326 (7.7)	12	5.2 (2.9-9.0)	11	4.7 (2.6-8.4)	621 (2.4)	3	4.8 (1.6-13.7)	0	0 (0.0-6.1)	.41	.61	<.001	<.001
Meets PA insurance coverage criteria, secondary analysis^f^^,^^h^														
No	22 815 (91.6)	125	5.5 (4.6-6.6)	18	0.8 (0.5-1.3)	32 108 (98.4)	180	5.6 (4.9-6.4)	14	0.4 (0.3-0.7)	.92	.99	.22	.20
Yes	2081 (8.4)	12	5.8 (1.9-16.7)	10	4.8 (2.6-8.9)	523 (1.6)	3	5.7 (1.9-16.7)	0	0 (0.0-7.3)	.39	.85	<.001	<.001

Abbreviations: BCRAT, Breast Cancer Risk Assessment Tool; BI-RADS, Breast Imaging and Reporting Data System; CDR, cancer detection rate; FN, false negative; ICR, interval cancer rate; NA, not applicable; TP, true positive.

^a^ Cancers among women with positive mammogram.

^b^ Calculated as TPs/total mammograms.

^c^ Cancers among women with negative mammograms.

^d^ Calculated as FNs/total mammograms.

^e^ Age was not statistically significantly different between Black and White women (*P* = .82).

^f^ Based on breast density and lifetime risk of breast cancer risk using the Gail model.

^g^ Participants with missing BCRAT scores were considered not eligible.

^h^ Participants with missing BCRAT scores were excluded.

Fewer Black women had extremely dense breasts based on BI-RADS categorization compared to White women (561 [2.1%] vs 1464 [5.8%]; *P* = .02). A smaller proportion of Black women (257 [0.7%] vs 1905 [6.4%]; *P* = .04) had greater than 20% BRCAT lifetime risk of breast cancer. Given these characteristics, Black women were less likely than white women to be eligible for supplemental screening under Pennsylvania law (523 [1.6%] vs 2081 [8.4%]; *P* = .02). A moderate number of women (10 951 [16.0%]) were missing BRCAT scores in the electronic health record, with White women having higher rates of missingness. After adjusting for age and BI-RADS density, White women were 11 times more likely to be eligible for supplemental screening under Pennsylvania law compared with Black women when missing BCRAT values were excluded (odds ratio, 11.32; 95% CI, 8.79-14.94; *P* = .001). We observed similar findings when women with missing BCRAT values were considered low risk in our analytic population. A Black woman, age 45 years, with heterogeneously dense breasts had approximately a 1% probability of being eligible. A White woman, age 45 years, with heterogeneously dense breasts had approximately a 12% probability of being eligible. These results were similar when stratified by pre– and post–COVID-19 years (eTables 2 and 3 in Supplement 1).

In this context, 100% sensitivity would mean that among women with a false-negative mammogram, all were considered eligible for supplemental screening. Conversely, 100% specificity would mean that among women with a true-negative mammogram, all were considered not eligible for supplemental screening. Eligibility criteria based on Pennsylvania law yielded lower sensitivity and higher specificity for detection of false-negative mammograms for breast cancer among Black women compared with White women (Table 2). When including women with missing BCRAT as not eligible, White women had a sensitivity of 29% (95% CI, 14%-45%) and a specificity of 93% (95% CI, 92%-93%) while Black women still had a sensitivity of 0% (95% CI, 0%-20%) and a specificity of 98% (95% CI, 98%-99%) for detecting women who had a cancer diagnosis within 1 year of their negative mammogram, with statistically significant differences for both sensitivity (*P* = .04) and specificity (*P* = .02) (Table 2). Excluding patients missing BCRAT scores yielded similar results (eTable 1 in Supplement 1).

**Table 2.** Sensitivity and Specificity of Insurance Coverage Criteria Based on Pennsylvania Eligibility Criteria for False-Negative Breast Cancer^a^

Eligibility	White women					Black women				
	Negative		Total	% (95% CI)		Negative		Total	% (95% CI)	
	False	True		Sensitivity	Specificity	False	True		Sensitivity	Specificity
Eligible	10	1786	1797	NA	NA	0	456	456	NA	NA
Not eligible	24	22 529	22 553	NA	NA	17	29 333	29 350	NA	NA
Total	35	24 315	24 350	29 (13.8-45.0)^b^	93 (92.3-93.0)^c^	17	29 789	29 806	0 (0.0-19.6)^b^	98 (98.3-98.6)^c^

Abbreviation: NA, not applicable.

^a^ Eligibility based on breast density among women with a nonactionable mammogram and available risk score. Patients with no risk score were considered low risk.

^b^ *P* = .04.

^c^ *P* = .02.

Using BI-RADS breast density categories of heterogeneously and extremely dense alone led to higher sensitivity and lower specificity among both Black and White women compared with using the eligibility criteria (Table 3). White women had sensitivity of 74% (95% CI, 57%-87%) and specificity of 60% (95% CI, 24%-80%) for false negatives, while Black women had a sensitivity of 53% (95% CI, 29%-77%) and a specificity of 80% (95% CI, 48%-96%) for false negatives, but with no statistically significant differences for either sensitivity (*P* = .20) or specificity (*P* = .61)

**Table 3.** Sensitivity and Specificity of Dense Breasts for False-Negative Breast Cancer^a^

Eligibility	White women					Black women				
	Negative, No.		Total, No.	% (95% CI)		Negative, No.		Total, No.	% (95% CI)	
	False	True		Sensitivity	Specificity	False	True		Sensitivity	Specificity
Eligible	26	9660	9686	NA	NA	9	5819	5828	NA	NA
Not eligible	9	14 655	14 664	NA	NA	8	23 970	23 978	NA	NA
Total	35	24 315	24 350	74 (56.8-86.9)^b^	60 (23.5-80.1)^c^	17	29 789	29 806	53 (28.7-77.1)^b^	80 (47.9-95.7)^c^

Abbreviation: NA, not applicable.

^a^ Among women with a nonactionable mammogram, available risk score, and heterogeneously or extremely dense breasts.

^b^ *P* = .20.

^c^ *P* = .61.

## Discussion

In our large population of women without a history of breast cancer undergoing screening mammography, Black women were less likely to have extremely dense breasts based on BI-RADS categorization (2.1% vs 5.8%) and had lower BCRAT lifetime risk estimates (0.7% vs 6.4%) than White women, making them less likely to be eligible for supplemental screening under recently passed Pennsylvania law. The law eliminates copays, deductibles, or co-insurance for supplemental screening, such as breast MRI and ultrasonography, for women meeting high risk criteria.^6^ However, if the eligibility criteria for supplemental screening based on breast density and BCRAT risk were applied, none of the Black women who had a false-negative mammogram would have been eligible for supplemental screening, compared with 29% of White women with a false-negative mammogram. Using BI-RADS pooled breast density categories without lifetime risk score (ie, extremely or heterogeneously dense) for supplemental screening would increase sensitivity for false-negative mammograms but would result in a very large number of MRIs or ultrasonography scans for both Black and White women.

While evidence is accumulating that supplemental screening can help reduce the risk of an interval cancer among women with dense breasts,^4^ our findings highlight potential difficulties in implementing breast density supplemental screening criteria to minimize false-negative mammograms at the population level and among Black women. Our results highlight that targeted supplemental screening based on BI-RADS breast density and the BCRAT risk scores fails to identify most false-negative first-round screening mammograms. These criteria perform even worse among Black women compared with White women.

Our results suggest that expanding supplemental screening eligibility to all women with heterogeneously or extremely dense breasts would identify more false negatives. However, given that approximately half of screening-aged women have dense breasts—most of whom will never develop breast cancer—offering supplemental screening to all would greatly increase the number of MRIs or ultrasonographic screenings performed.^14^ This expansion would lead to high costs and limited, delayed access for those who need it most, making this approach currently unfeasible due to capacity constraints.

Insurance coverage laws substantially affect patients and payers, so evaluating and tailoring these policies is crucial to improving early breast cancer detection. Future research should look at similar outcomes with larger sample sizes to assess the risks and benefits of supplemental screening mandates. While breast MRI is sensitive, it also causes many false-positive biopsies, imposing psychological and financial burdens.^15^ More precise methods to assess false-negative risk could enable targeted supplemental screening, maximizing benefits and minimizing harms.

Our results confirm previous literature demonstrating that Black women have lower rates of heterogeneously or extremely dense breasts using the qualitative BI-RADS density assessments compared with White women.^16,17,18,19,20^ Other studies have also confirmed that the use of BI-RADS density alone has resulted in modest improvement in risk stratification approaches.^21,22^ Given that Black women have lower prevalence of dense breasts by BI-RADS assessments, the new supplemental screening law is unlikely to meaningfully reduce the marked racial disparities in breast cancer mortality.

Reducing the risk of false-negative mammograms and improving early breast cancer detection is especially important for high-risk women. The DENSE trial demonstrated a 50% reduction in interval cancer rates with MRI screening. However, this study used a 2-year screening interval, and its findings may not fully apply to the US population.^4^ Still, US-based observational studies show similar cancer detection rates in women with dense breasts, suggesting that MRI identifies cancers that mammography alone may miss.^23^

In our analysis, Black women were less likely to have false-negative mammograms compared with White women, even among those with dense breasts. This finding contrasts with recent research showing that Black women experience the highest rates of false negatives among racial and ethnic groups.^24^ One possible explanation is that newer BI-RADS density classifications—which emphasize how dense tissue can obscure cancers—may not fully capture the risk of false negatives. Quantitative measures, such as dense volume, may offer more accurate insight.

Another consideration is that BI-RADS density may not effectively predict the risk of aggressive cancers. Black women are at higher risk for these cancers, and current models may overlook key factors. For instance, BMI has been linked to an increased risk of advanced breast cancer diagnosis within 2 years of a negative mammogram (C. Vachon, PhD, unpublished data, 2025).^25^ The updated Breast Cancer Surveillance Consortium (BCSC) version 3 model significantly improved risk prediction after incorporating BMI, with the greatest gains observed in Black women.^26^

Future studies should evaluate whether combining BCSC version 3 with BMI, race and ethnicity, and breast density can better identify candidates for supplemental screening—and whether this leads to earlier or more accurate diagnoses. In addition, other tools, such as biomarkers or artificial intelligence, may help reduce false negatives where MRI screening alone falls short.

Finally, if fewer Black women are being recommended for supplemental screening, this may reflect an effort to reduce unnecessary harm—particularly if additional screening does not significantly improve survival or detection rates. Ultimately, screening guidelines must balance benefits with potential harms, and more research is needed to refine strategies that ensure equitable, evidence-based care tailored to individual risk profiles.

### Strengths and Limitations

This study had strengths and limitations. A major strength of our study is the large number of screening studies from both Black and White women at a large tertiary hospital system. The study also has several limitations.

We only considered the Pennsylvania eligibility criteria for supplemental screening based on BI-RADS breast density and BCRAT lifetime risk (which may underestimate risk in Black women), as the information on other criteria, such as genetic predisposition to breast cancer or personal and family history of breast cancer, had higher rates of missingness. Unmeasured confounding may be a source of bias in our results because we did not include other breast cancer risk factors such as BMI, menopausal status, or age at menarche as adjustment factors in our analysis with eligibility as the outcome. BMI was not included as an adjustment factor in this analysis because the Pennsylvania law does not consider BMI when screening high-risk women. To ensure that our analysis aligns with the provisions of the new law, we chose not to incorporate it as a variable. A limitation of this study is that it only includes one mammogram per patient. Across multiple rounds of screening, more patients with false-negative studies could be identified, and additionally, cancers that would have been screen detected at the screening round could be identified earlier with supplemental screening. However, there is limited evidence in the literature to suggest that this approach would effectively reduce late-stage diagnoses.^27^

We acknowledge that a low percentage of women who qualify for supplemental screening receive it. One potential reason is insurance barriers, specifically as supplemental breast MRI screening coverage varies by state. A growing number of states, including Pennsylvania, require insurance coverage of supplemental screening for women with dense breasts. The purpose of this study was to evaluate the eligibility criteria in Pennsylvania, and whether those criteria successfully identified women with subsequent false-negative mammograms. While the Pennsylvania law went into effect on January 1, 2022, copays, co-insurance, and/or deductibles may still be charged and may render MRI cost-prohibitive. Pennsylvania passed a second law eliminating out-of-pocket costs for supplemental screening, which took full effect on January 1, 2025. While the current study focuses on eligibility criteria, subsequent research is needed to investigate the extent to which these new laws will affect MRI utilization. Future studies should investigate the impact of insurance coverage alone on utilization of supplemental breast MRI screening among women with dense breasts.^28^ We focused on the Gail risk prediction tool because of its availability in our electronic health record. Additionally, we lacked sufficient risk factors to use the Tyrer-Cuzick model. However, because existing risk models have only moderate accuracy we do not expect model choice to have a large impact on accuracy of risk assessment, particularly in a general screening population. We were only able to explore the supplemental screening law within one urban tertiary health care system, which may limit the generalizability of the study. However, this is the study that we know of to explore potential outcomes of Pennsylvania’s law mandating insurance coverage for supplemental breast cancer screening among Black and White women. Asian women were not included in the current study, as our screening population includes few Asian patients. While the study period includes the beginning of the COVID-19 pandemic, only a small portion of the mammograms studied were during that period. When conducting a stratified analysis by years pre– and post–COVID-19, the results were similar, suggesting that the COVID-19 pandemic did not lead to a significant change in the outcome of patients undergoing mammogram in our population (eTables 2-3 in Supplement 1). However, we cannot definitively rule out the possibility that pandemic-related changes in health care access or utilization had an impact on our results.

## Conclusions

Our cross-sectional study found that if BI-RADS breast density and the Gail model are used to determine eligibility for supplemental screening, Black women were less likely to qualify, despite their greater risk of breast cancer death. Improved current approaches as well as exploration of novel risk factors are needed to identify Black women at risk for false-negative mammograms who might benefit from supplemental screening. There is a need for risk models that more accurately estimate risk for Black women, including existing models that could incorporate quantitative measures of density or other novel biomarkers, using artificial intelligence algorithms that can reduce false-negative mammograms. We acknowledge that disparities in breast cancer mortality are complex and that successfully addressing each disparity will require multiple interventions with concerted efforts from lawmakers, lobbyists, scientists, and clinicians. The recent legislation was not intended to address racial disparities in breast cancer specifically; however, proactive evaluation of the potential impacts of new policies on various patients populations is needed to help prevent new practices from inducing and/or exacerbating existing disparities in breast cancer, mainly as Black women are less likely to qualify for supplemental screening, despite their greater risk of breast cancer diagnosis and death using the BI-RADS breast density definitions.

## Notes

Supplement 1.
eMethods.
**eTable 1.** Sensitivity and Specificity of Insurance Coverage Criteria Based on Pennsylvania Eligibility Criteria for False-Negative Breast Cancer (Excluding Patients With Missing BCRAT) **eTable 2.** Breast Density, BCRAT Lifetime Risk, and Eligibility for Insurance Coverage of Supplemental Screening in Pennsylvania Among Women With Mammography Screening at the Hospital of the University of Pennsylvania, 2015-2019 **eTable 3.** Breast Density, BCRAT Lifetime Risk, and Eligibility for Insurance Coverage of Supplemental Screening in Pennsylvania Among Women With Mammography Screening at the Hospital of the University of Pennsylvania, 2020-2021

Supplement 2.
Data Sharing Statement
