# Supplementary material for: Racial Differences in Screening Eligibility by Breast Density After State-Level Insurance Expansion
Source: JAMA Netw Open. 2025 Aug 5;8(8):e2525216. doi: 10.1001/jamanetworkopen.2025.25216 (PMC12326280; doi:10.1001/jamanetworkopen.2025.25216)
Supplement: Supplement 1. — eMethods. eTable 1. Sensitivity and Specificity of Insurance Coverage Criteria Based on Pennsylvania Eligibility Criteria for False-Negative Breast Cancer (Excluding Patients With Missing BCRAT) eTable 2. Breast Density, BCRAT Lifetime Risk, and Eligibility for Insurance Coverage of Supplemental Screening in Pennsylvania Among Women With Mammography Screening at the Hospital of the University of Pennsylvania, 2015-2019 eTable 3. Breast Density, BCRAT Lifetime Risk, and Eligibility for Insurance Coverage of Supplemental Screening in Pennsylvania Among Women With Mammography Screening at the Hospital of the University of Pennsylvania, 2020-2021 [file jamanetwopen-e2525216-s001.pdf]

# Supplemental Online Content

Mahmoud MA, Ehsan S, Ginzberg SP, et al. Racial differences in screening eligibility by breast density after state-level insurance expansion. *JAMA Netw Open.* 2025;8(8):e2525216. doi:10.1001/jamanetworkopen.2025.25216

**eMethods.**

**eTable 1.** Sensitivity and Specificity of Insurance Coverage Criteria Based on Pennsylvania Eligibility Criteria for False-Negative Breast Cancer (Excluding Patients With Missing BCRAT)

**eTable 2.** Breast Density, BCRAT Lifetime Risk, and Eligibility for Insurance Coverage of Supplemental Screening in Pennsylvania Among Women With Mammography Screening at the Hospital of the University of Pennsylvania, 2015-2019

**eTable 3.** Breast Density, BCRAT Lifetime Risk, and Eligibility for Insurance Coverage of Supplemental Screening in Pennsylvania Among Women With Mammography Screening at the Hospital of the University of Pennsylvania, 2020-2021

This supplemental material has been provided by the authors to give readers additional information about their work.

**eMethods.**

We conducted a retrospective cross-sectional analysis among women without prior breast cancer or known *BRCA1/2* mutations who underwent routine screening mammography, completed risk factor questionnaires from January 2015 to December 2021, and had a full year of follow-up to focus our study on women with dense breasts (Figure 1). Informed consent was waived in this secondary analysis of data obtained during routine clinical care. The study was HIPAA-compliant and approved by the Institutional Review Board of the University of Pennsylvania, and a waiver of informed consent was granted for this review of existing clinical data. The Strengthening the Reporting of Observational Studies in Epidemiology (STROBE) checklist was reviewed, and the manuscript was drafted in accordance with these guidelines.[8]

Our study population included Black or White women (including Hispanic women) based on self-reported race noted in the electronic health record, between the ages of 40 and 74, for a total of 68,478 women. For each mammogram, breast density was characterized according to American College of Radiology BI-RADS density categories: almost entirely fatty, scattered areas of fibroglandular density, heterogeneously dense, and extremely dense. According to PA law, women can be considered eligible for supplemental screening if they are high risk as defined by several criteria (Table 1). For our study, we focused on eligibility criteria based on breast density or Gail risk >20% among women with no personal history of breast cancer. We defined women as meeting eligibility criteria if they had 1) extremely dense breasts *OR* 2) heterogeneously dense breasts based on BI-RADS categorization *and* greater than a 20% lifetime risk of breast cancer. Lifetime risk was estimated using the Breast Cancer Risk Assessment Tool (BCRAT).[9]

A false-negative mammogram was defined as a cancer diagnosis within one year of a non-actionable screening mammogram (initial BI-RADS Assessment Negative [1] or Benign [2]). A true-positive mammogram was calculated as a cancer diagnosis within one year of a positive mammogram (initial screening BI-RADS Assessment 0 with final BI-RADS at diagnostic recall assessments 3, 4 or 5). The

cancer detection rate (CDR) was defined as the number of true-positive mammograms per total number of mammograms. The interval cancer rate was defined as the number of false-negative mammograms among the total number of mammograms. Both rates were expressed per 1000 screening mammograms.[10] We report the performance metrics (true-positive [TP], CDR, false-negative [FN], and interval cancer rate) overall, by density, lifetime risk, insurance coverage, stratified by race, as typically done using benchmark guidelines in the Breast Cancer Surveillance Consortium.[29,30]

Characteristics of Black and White women were summarized by frequency and percentage or mean and standard deviation values of BI-RADS density, age, and eligibility for insurance coverage. We also examined the sensitivity and specificity of both insurance coverage criteria based on the PA law (BI-RADS Heterogeneously Dense & BCRAT >20% OR BI-RADS Extremely Dense), as well as using BI-RADS breast density alone (heterogeneously or extremely dense breasts vs. entirely fatty or scattered fibroglandular tissue) with respect to false-negative breast cancer among women with negative mammograms.

We tested for associations of eligibility according to the PA law and risk of a false-negative mammogram, adjusting for age and BI-RADS density and stratified by race, among women with negative mammograms, using logistic regression. We also tested the association between race and the odds of eligibility, adjusted for age and BI-RADS density. For each analysis, women with missing BCRAT lifetime risk percentages were considered low risk, reflecting how they would be clinically treated (primary analysis), and a complete case analysis was conducted with women who had an available BCRAT score (secondary analysis). As a sensitivity analysis, we stratified the analysis by pre and post COVID 19 years (2015-2019 vs 2020-2021). Statistical tests were two-sided with an alpha level of 0.05 considered significant. All data analyses and management were performed using RStudio, version 1.4.1106 (R Foundation for Statistical Computing, Vienna, Austria).

**eTable 1. Sensitivity and Specificity of Insurance Coverage Criteria Based on Pennsylvania Eligibility Criteria[1] for False-Negative Breast Cancer (Excluding Patients With Missing BCRAT)**

| | White | | | | | Black | | | | |
|---|---|---|---|---|---|---|---|---|---|---|
| | False Negative | True Negative | TOTAL | Sensitivity | Specificity | False Negative | True Negative | TOTAL | Sensitivity | Specificity |
| Eligible | 10 | 2061 | 2071 | | | 0 | 523 | 523 | | |
| Not Eligible | 18 | 22797 | 22815 | | | 14 | 32,094 | 32,108 | | |
| Total | 28 | 24858 | 24886 | 35.7% (95% CI: 18.6–55.9%) *p*= 0.03 | 91.7% (95% CI: 91.0–92.4%) *p*=0.01 | 14 | 32,617 | 32,631 | 0% (95% CI: 0.0–21.5%) | 98.4% (95% CI: 98.1–98.6%) |

[1]eligibility based on breast density among women with a non-actionable mammogram and available BCRAT score and considering patients with no BCRAT score as low risk

| **eTable 2.** Breast Density, BCRAT Lifetime Risk, and Eligibility for Insurance Coverage of Supplemental Screening in Pennsylvania Among Women With Mammography Screening at the Hospital of the University of Pennsylvania, 2015-2019 | | | | | | | | | | | |
|---|---|---|---|---|---|---|---|---|---|---|---|
| | **All (N=50,729)** | | | | | | | | | | |
| | **White (N=22,811 women)** | | | | | **Black (N=27,918 women)** | | | | | **P values comparing metrics by Race** |
| | **N (%)** | **TP[1]** | **CDR[2] Per 1000 (95% CI)** | **FN[3]** | **Int[4] Per 1000 (95% CI)** | **N (%)** | **TP[1]** | **CDR[2] Per 1000 (95% CI)** | **FN[3]** | **Int[4] Per 1000 (95% CI)** | |
| **Total Screens** | 22,811 (100) | 116 | 5.1 (4.8-5.4) | 30 | 1.3 (1.0-1.6) | 27,918 | 157 | 5.6 (5.3-5.9) | 12 | 0.4 (0.3-0.6) | TP: 0.57, CDR: 0.49 FN: 0.02, Int: 0.02 |
| **Age (median, Interquartile Range [IQR], yrs)** | 58 (16) | -- | -- | -- | -- | 57 (15) | -- | -- | -- | -- | 0.82 |
| **BI-RADS Density** | | | | | | | | | | | |
| Almost entirely fatty | 1,478 (6.5) | 4 | 2.7 (1.5-4.7) | 0 | 0 (0.0-3.5) | 4,589 (16.4) | 12 | 2.6 (1.7-3.9) | 0 | 0 (0.0-5.1) | TP: 0.12, CDR: 0.08 FN/Int: 0.87 |
| Scattered fibroglandular | 12,287 (54.0) | 78 | 6.35 (5.9-6.6) | 8 | 0.7 (0.4-1.0) | 17,861 (64.0) | 112 | 6.27 (5.9-6.6) | 5 | 0.3 (0.2-0.4) | TP: 0.21, CDR: 0.31, FN: 0.52, Int: 0.41 |
| Heterogeneously dense | 8,049 (35.3) | 33 | 4.1 (3.-5.0) | 18 | 2.2 (1.8-2.7) | 5,095 (18.2) | 32 | 6.3 (5.8-6.8) | 7 | 1.4 (1.1-1.8) | TP: 0.14, CDR: 0.28, FN: 0.03, Int: 0.05 |
| Extremely dense | 996 (4.4) | 1 | 1 (0.2-2.9) | 4 | 4.0 (1.3-7.5) | 373 (1.3) | 1 | 2.7 (1.0-5.9) | 0 | 0 (0.0-2.9) | TP: <0.001, CDR: <0.001 FN: <0.001, Int: <0.001 |
| **>20% lifetime risk based on the BCRAT Model** | | | | | | | | | | | |
| No | 17,678 (77.5) | 83 | 4.7 (4.2-5.3) | 17 | 1.0 (0.7-1.4) | 24,020 (86) | 135 | 5.6 (5.3-5.9) | 10 | 0.4 (0.3-0.6) | TP: 0.43, CDR: 0.66, FN: 0.13, Int: 0.12 |
| Yes | 1,532 (6.7) | 14 | 9.1 | 6 | 3.9 (1.9-6.8) | 201 (0.7) | 1 | 5.0 | 0 | 0 (0.0-1.8) | TP: 0.03, CDR: 0.03, |

© 2025 Mahmoud MA et al. *JAMA Network Open*.

| | | | | | | | | | | |
|---|---|---|---|---|---|---|---|---|---|---|
| | | | (6.1-13.4) | | | | | (0.0-14.8) | | | FN: <0.001, Int: <0.001 |
| Missing BCRAT Score | 5,185 (17.2) | 32 | 6.2 (4.4-8.7) | 3 | 0.6 (0.2-1.8) | 5,766 (15.0) | 31 | 5.4 (3.8-7.6) | 7 | 1.2 (0.6-2.5) | TP: 0.75, CDR: 0.64 FN: 0.14, Int: 0.15 |
| **Meets PA insurance coverage criteria (Missing BCRAT Score = not eligible)[5]** | | | | | | | | | | | |
| No | 21,120 (93) | 107 | 5.1 (4.7-5.4) | 23 | 1.1 (0.8-1.5) | 27,501 (99) | 155 | 5.6 (5.3-6.0) | 12 | 0.4 (0.3-0.6) | TP: 0.88, CDR: 0.95, FN: 0.19, Int: 0.12 |
| Yes | 1,690 (7) | 9 | 5.3 (4.2-6.7) | 7 | 5.1 (3.6-7.3) | 417 (1) | 2 | 4.8 (2.7-7.9) | 0 | 0 (0.0-4.3) | TP: 0.75, CDR: 0.75, FN: 0.12, Int: 0.20 |
| **Meets PA insurance coverage criteria (Missing BCRAT Score excluded)[5]** | | | | | | | | | | | |
| No | 17,677 (92) | 88 | 5.0 (4.6-5.4) | 17 | 1.0 (0.7-1.4) | 23, 862 (99) | 134 | 5.6 (5.3-6.0) | 10 | 0.4 (0.3-0.6) | TP: 0.92, CDR: 0.99, FN: 0.22, Int: 0.20 |
| Yes | 1,532 (8) | 9 | 5.9 (4.5-7.6) | 6 | 3.9 (2.2-6.3) | 359 (1) | 2 | 5.6 (4.0-7.7) | 0 | 0 (0.0-5.1) | TP: 0.70, CDR: 0.70, FN: 0.12, Int: 0.32 |

[1]True Positives (Cancers among women with positive mammogram); [2]Cancer Detection Rate per 1,000 (TP/total mammograms); [3]False Negatives (Cancers among women with negative mammograms); [4]Interval cancer rate per 1,000 (FN/total mammograms); - = Not Applicable
[5]Based on breast density and lifetime risk of breast cancer risk using the Gail model

© 2025 Mahmoud MA et al. *JAMA Network Open*.

| eTable 3. Breast Density, BCRAT Lifetime Risk, and Eligibility for Insurance Coverage of Supplemental Screening in Pennsylvania Among Women With Mammography Screening at the Hospital of the University of Pennsylvania, 2020-2021 | | | | | | | | | | | |
|---|---|---|---|---|---|---|---|---|---|---|---|
| | **All (N=17,749)** | | | | | | | | | | |
| | **White (N=7,270 women)** | | | | | **White (N=7,270 women)** | | | | | **P values comparing metrics by Race** |
| | **N (%)** | **TP[1]** | **CDR[2] Per 1000 (95% CI)** | **FN[3]** | **Int[4] Per 1000 (95% CI)** | **N (%)** | **TP[1]** | **CDR[2] Per 1000 (95% CI)** | **FN[3]** | **Int[4] Per 1000 (95% CI)** | |
| **Total Screens** | 7,270 (100) | 52 | 7.2 (6.0-8.5) | 5 | 0.7 (0.5-1.0) | 10,479 (100) | 58 | 5.5 (5.0-6.1) | 5 | 0.5 (0.3-0.7) | TP: 0.67, CDR: 0.27, FN: 0.09, Int: 0.42 |
| **Age (median, Interquartile Range [IQR], yrs)** | 57 (16) | -- | -- | -- | -- | 57 (15) | -- | -- | -- | -- | 0.82 |
| **BI-RADS Density** | | | | | | | | | | | |
| Almost entirely fatty | 301 (4.1) | 0 | 0 (0.0-5.4) | 0 | 0 (0.0-4.8) | 1,183 (11.3) | 6 | 5.07 (4.2-6.1) | 0 | 0 (0.0-6.3) | TP: 0.18, CDR: 0.39, FN: 0.51, Int: 0.35 |
| Scattered fibroglandular | 3,575 (49.2) | 28 | 7.8 (7.2-8.5) | 1 | 0.3 (0.1-0.6) | 6,802 (64.9) | 38 | 5.6 (5.0-6.2) | 3 | 0.4 (0.3-0.7) | TP: 0.29, CDR: 0.12, FN: 0.55, Int: 0.12 |
| Heterogeneously dense | 2,927 (40.3) | 22 | 7.5 (6.3-8.9) | 1 | 0.3 (0.2-0.6) | 2,306 (22.0) | 13 | 5.6 (4.8-6.6) | 2 | 0.9 (0.5-1.5) | TP: 0.69, CDR: 0.28, FN: 0.52, Int: 0.71 |
| Extremely dense | 467 (6.4) | 2 | 4.3 (2.2-7.9) | 3 | 6.4 (4.6-8.6) | 188 (1.8) | 1 | 5.3 (3.1-8.9) | 0 | 0 (0.0-4.8) | TP: 0.53, CDR: 0.80, FN: 0.62, Int: 0.37 |
| **>20% lifetime risk based on the BCRAT Model** | | | | | | | | | | | |
| No | 5,313 (73.1) | 38 | 7.2 (6.1-8.3) | 3 | 0.6 (0.3-0.9) | 8,354 (79.7) | 47 | 5.6 (5.1-6.2) | 4 | 0.5 (0.3-0.7) | TP: 0.29, CDR: 0.73, FN: 0.23, Int: 0.25 |
| Yes | 373 (5.13) | 2 | 5.36 (2.4-10.7) | 2 | 5.4 (2.4-9.9) | 56 (0.53) | 0 | 0 (0.0-3.2) | 0 | 0 (0.0-5.1) | TP: 0.69, CDR: 0.87, FN: 0.23, Int: 0.33 |

© 2025 Mahmoud MA et al. *JAMA Network Open*.

| | | | | | | | | | | |
|---|---|---|---|---|---|---|---|---|---|---|
| Missing BCRAT Score | 1,584 (21.8) | 12 | 7.6 (5.9-9.6) | 0 | 0 (0.0-2.3) | 2,069 | 11 | 5.3 (4.6-6.1) | 1 | 0.5 (0.3-0.9) | TP: 0.76, CDR: 0.57, FN: 0.12, Int: 0.18 |
| **Meets PA insurance coverage criteria (Missing BCRAT Score = not eligible)[5]** | | | | | | | | | | | |
| No | 6,634 (91) | 49 | 7.4 (6.2-8.6) | 1 | 0.2 (0.0-0.5) | 10,275 (98) | 57 | 5.6 (5.0-6.2) | 5 | 0.5 (0.3-0.7) | TP: 0.89, CDR: 0.87, FN: 0.19, Int: 0.22 |
| Yes | 636 (9) | 3 | 4.7 (2.5-8.9) | 4 | 6.4 (3.1-10.4) | 204 (2) | 204 | 4.9 (2.8-8.3) | 0 | 0 (0.0-4.6) | TP: 0.92, CDR: 0.88, FN: 0.12, Int: 0.31 |
| **Meets PA insurance coverage criteria (Missing BCRAT Score excluded)[5]** | | | | | | | | | | | |
| No | 5,137 (90) | 37 | 7.2 (6.3-8.3) | 1 | 0.2 (0.1-0.5) | 8,246 (98) | 46 | 5.6 (5.0-6.2) | 4 | 0.5 (0.3-0.7) | TP: 0.89, CDR: 0.85, FN: 0.18, Int: 0.30 |
| Yes | 549 (10) | 3 | 5.5 (2.6-11.5) | 4 | 7.3 (3.6-12.8) | 164 (2) | 1 | 6.1 (2.9-12.8) | 0 | 0 (0.0-5.1) | TP: 0.75, CDR: 0.75, FN: 0.12, Int: 0.32 |

[1]True Positives (Cancers among women with positive mammogram); [2]Cancer Detection Rate per 1,000 (TP/total mammograms); [3]False Negatives (Cancers among women with negative mammograms); [4]Interval cancer rate per 1,000 (FN/total mammograms); - = Not Applicable
[5]Based on breast density and lifetime risk of breast cancer risk using the Gail model

© 2025 Mahmoud MA et al. *JAMA Network Open*.
