# Supplementary material for: Racial Differences in Screening Eligibility by Breast Density After State-Level Insurance Expansion
Source: JAMA Netw Open. 2025 Aug 5;8(8):e2525216. doi: 10.1001/jamanetworkopen.2025.25216 (PMC12326280; doi:10.1001/jamanetworkopen.2025.25216)
Supplement: Supplement 2. — Data Sharing Statement [file jamanetwopen-e2525216-s002.pdf]

Data Sharing Statement

Mahmoud. Racial Differences in Screening Eligibility by Breast Density After State-Level Insurance Expansion. *JAMA Netw Open*. Published August 05, 2025. doi:10.1001/jamanetworkopen.2025.25216

Data
Data available: No

Additional Information
Explanation for why data not available: data has patient identifiers attached to it
